# Application study of 3D LAVA-Flex on lumbar intervertebral disc degeneration

**DOI:** 10.1186/s40001-021-00512-y

**Published:** 2021-05-07

**Authors:** Tiefang Liu, Yonghao Wang, Zhengyang Xu, Tao Wu, Xiao Zang, Meng Li, Jinfeng Li

**Affiliations:** 1 Department of Radiology, The First Medical Center of PLA General Hospital, Beijing, 100853 China; 2 Department of Ultrasound, The Eighth Medical Center of PLA General Hospital, Beijing, 100091 China; 3 MR Enhanced Application Team, GE Healthcare, Beijing, 100176 China

**Keywords:** 3D LAVA-Flex, Magnetic resonance imaging, Disc degeneration, Virtual endoscopy

## Abstract

**Background:**

Degeneration of the intervertebral discs are very common diseases, indicating the specific or malignant changes in intervertebral disc component, structure and function. Imaging examination is currently used to evaluate the severity of lumbar intervertebral disc degeneration. This study was designed to investigate the diagnostic value of 3D LAVA-Flex in lumbar intervertebral disc degeneration.

**Material and methods:**

Sagittal 3D LAVA-Flex and T2WI scans were performed in 45 patients with lumbar intervertebral disc degeneration. On T2WI, the degenerated intervertebral disc in every patient was evaluated using Pfirrmann grade. Then, the patients were re-evaluated using 3D LAVA-Flex with considerations of the distinction of nucleus pulposus and annulus fibrosus, hypointense signal of intervertebral disc and height of intervertebral disc. The evaluation results were compared between 3D LAVA-Flex and T2WI. Virtual endoscopy was also performed to evaluate the degenerated intervertebral disc.

**Results:**

The intermediate–intense signal of nucleus pulposus and complete ring-shaped hyperintense signal of annulus fibrosus were found and the distinction of nucleus pulposus and annulus fibrosus was clear in the normal intervertebral disc on 3D LAVA-Flex. The incidence of linear hypointensity of narrowed intervertebral space (65/91) was higher than that of normal intervertebral space (4/134) (*P* = 0.000). A good consistency was shown between the LAVA-Flex grade and T2WI-based Pfirrmann grade. Virtual endoscopy based on 3D LAVA-Flex could help clearly show the anatomic relationship between the degenerated disc and intervertebral foramen.

**Conclusions:**

3D LAVA-Flex and T2WI show similar efficacy in evaluating lumbar intervertebral disc degeneration. 3D LAVA-Flex-based virtual endoscopy possesses great potential in the study of intervertebral disc abnormalities.

## Background

Degeneration and prolapse of lumbar intervertebral disc and stenosis of lumbar intervertebral foramen are very common diseases [1]. The severity of these disorders is associated with many factors. Surgery should be performed at the time at which clinical symptoms correspond to surgical indication. Imaging examination is currently used to validate clinical judgment, exclude intervertebral disc-unrelated disorders, and ensure a successful surgery [2, 3]. No imaging quantitative indices and golden standards have been reported to evaluate the severity of lumbar intervertebral disc degeneration and related surgical indications.

Magnetic resonance imaging (MRI) plays an important role in the diagnosis and differential diagnosis of lumbar intervertebral disc and spine diseases and is of important clinical value for evaluating the severity of lumbar intervertebral disc degeneration and spinal stenosis [4, 5]. Conventional MRI techniques, including T1-weighted imaging (T1WI) and T2-weighted imaging (T2WI), have been used for diagnosis of lumbar spine diseases. The advance in computational neuroimaging techniques, for example the recently emerging three-dimensional liver imaging with volume acceleration-flexible (3D LAVA-Flex), virtual endoscopy, and diffusion tensor imaging (DTI), provides a possibility for precise quantitative evaluation of intervertebral disc degeneration and nerve root compression using region of interest-based analysis approaches [6, 7].

The evolutionary process from the earliest 2D fast spoiled gradient-recalled-echo (FSGPR) to 3D fast acquisition with multiphase EFGRE (FAME) to 3D liver acquisition volume acceleration (LAVA) betters contrast-enhanced abdominal MRI. LAVA provides 3D images with high temporal, spatial resolution and excellent homogeneous fat suppression, makes multiple arterial phase dynamic enhanced scans of the liver possible, and is competent to clinical application in the diagnosis of small lesions, such as bile duct lesion [8]. LAVA-Flex can provide a high-quality and very consummate dynamic contrast-enhanced abdominal scan. Compared to 2D FSPGR and 3D FAME, 3D LAVA-Flex provides higher temporal and spatial resolution because it has a unique k-space data filling technique [8]. Multiple arterial phase dynamic contrast-enhanced scans of the liver can help identify the type of blood supplied to the damaged liver, which is of importance for determining the nature of the lesion. Magnetic resonance cholangiopancreatography (MRCP) combined with 3D LAVA-Flex can help determine the presence of bile duct obstruction and the level of obstruction, thereby further identifying the cause of bile duct obstruction. This is very important for determining treatment protocols and judging the prognosis.

Although 3D LAVA-Flex dynamic contrast-enhanced scans can help provide a perfect abdominal scanning protocol, it has some drawbacks to be determined in the clinic. For example, it cannot well differentiate lipid substances with different components because the nature of lesion should be identified. 3D LAVA-Flex recently developed by GE Healthcare adds in-phase and opposed-phase sequence and precisely determines whether degenerated (immature) fat exists in the lesion or liver. As for space-occupying lesions of the liver, presence of fatty infiltration in the lesions is an important differential diagnosis of liver tumor. Although liver cancer and hepatic adenoma both exhibit fatty degeneration, the latter has a low incidence. Mature fat is of significance in the diagnosis of vascular smooth muscle lipoma or hamartoma. 3D LAVA-Flex can generate water-only, fat-only, in-phase and out-of-phase images in one single acquisition. Therefore, it can be used to differentiate mature and immature fat, which is of critical importance in qualitative diagnosis of lesions.

In addition to above advantages, 3D LAVA-Flex also has many other clinical advantages, including higher single-to-noise ratio, and greater effective diagnosis scope inside and between the layers. In clinical practice, 3D LAVA-Flex can generate multiple image contrasts in one single acquisition, provide a larger-area abdominal scan, and reject fat signal in the partial central zone. 2D FSPGR, a fast spoiled gradient-recalled-echo sequence, was previously used as a dynamic contrast-enhanced scanning technique. Its emergence initiates dynamic contrast-enhanced MRI scans of the abdomen. Homogeneous fat suppression is a key to acquiring good image contrast during contrast-enhanced scans of the liver. 2D FSPGR uses fat suppression by conventional frequency selection (chemical method), which limits scanning speed to a certain degree, so multiple phase dynamic contrast-enhanced scans cannot be performed using this scanning technique. In addition, isotropic reconstruction cannot be achieved by 2D image acquisition. Therefore, only one single acquisition cannot provide satisfactory information in the other perspectives. Although the emergence of 3D LAVA-Flex dynamic contrast-enhanced scanning technique changes the concept of dynamic contrast-enhanced scan of the abdomen, it faces some challenges, for example, fat suppression in the large-scale partial central zone. The use of special fat suppression technique determines that 3D LAVA-Flex images depend on magnetic field uniformity and signal-to-noise ratio to a certain degree. Greatly different from conventional dynamic contrast-enhanced scanning technique, 3D LAVA-Flex uses unique water–fat separation method rather than fat suppression pulse. 3D LAVA-Flex can generate water-only, fat-only, in-phase and out-of-phase images in one single acquisition, which fundamentally alters the procedure of abdominal examination [8].

3D LAVA-Flex provides higher temporal and spatial resolution. The most ideal situation is to achieve scans with higher spatial resolution while maintaining temporal resolution. Dynamic contrast-enhanced scan of the abdomen is generally performed under a breath-hold state, which brings difficulties for simultaneous consideration of both temporal and spatial resolution. A scan with short TR and TE can meet the dual demands of temporal and spatial resolution. However, a higher-performance gradient system is required to achieve shorter TR and TE. It should be noted that gradient performance is not a simple gradient engineering value but the largest gradient performance harvested in the clinic. As for a magnetic resonance imaging system, rapid three-dimensional dynamic contrast-enhanced scanning technique itself is not important, but more concerns have been paid to its application in evaluation of gradient performance.

This study aimed to evaluate annulus fibrosus and nucleus pulposus of degenerated intervertebral disc and grade intervertebral disc disorder using 3D LAVA-Flex, and investigate the anatomic relationship between protruded intervertebral disc and intervertebral foramen using virtual endoscopy.

## Material and methods

### Study population

Forty-five patients with low back pains, consisting of 25 males and 20 females, aged 23–66 years (mean 55.98 ± 11.98 years old) were included in this study. All patients had different degrees of intervertebral disc degeneration and different degrees of spinal stenosis. Written informed consent was obtained from each patient. The study was approved by the Ethics Committee of The First Medical Center of Chinese PLA General Hospital.

### MRI and image acquisition

3.0 T superconducting magnet (MR 750, GE Healthcare, Milwaukee, USA) with the maximum gradient strength of 40 mT/m and the maximum slew rate of 150 mT/m/ms; an 8-channel spine coil. For sagittal scan of the lumbar spine, T2WI scanning parameters include TR/TE = 2183/108 ms, layer thickness = 5 mm, interval = 1 mm, layer number = 22, FOV = 24 cm × 24 cm, matrix = 320 × 224, NEX = 1.0. The lumbar spine including the lumbosacral vertebrae was scanned using 3D FSPGR (LAVA-Flex) with the following parameters: TR/TE = 4/1.7 ms, layer thickness = 4 mm, interval = 0 mm, layer number = 32, FOV = 32 cm × 25.2 cm, matrix = 208 × 224, NEX = 1.0. All scans were performed by a professional radiologist and the subject’s body was asked to be in a static state when being scanned.

### Pfirrmann classification

The intervertebral discs on T2-weighted images were graded by Pfirrmann classification as follows [9]: grade I, homogeneous bright white hyperintense signal of the nucleus pulposus, clear distinction of nucleus pulposus and annulus fibrosus, and normal height of intervertebral disc; II, inhomogeneous bright white hyperintense signal of the nucleus pulposus, with or without horizontal, hypointense band, clear distinction of nucleus pulposus and annulus fibrosus, and normal height of intervertebral disc; III, inhomogeneous intermediate–intense (gray) signal of the nucleus pulposus, unclear distinction of nucleus pulposus and annulus fibrosus, normal to slightly decreased height of intervertebral disc; IV, inhomogeneous intermediate to hypointense (gray or black) signal of the nucleus pulposus, lost distinction of nucleus pulposus and annulus fibrosus and normal to moderately decreased height of intervertebral disc; V, inhomogeneous hypointense (black) signal of the nucleus pulposus, lost distinction of nucleus pulposus and annulus fibrosus and collapsed disc space.

### Imaging evaluation criteria

**Fig. 1:**
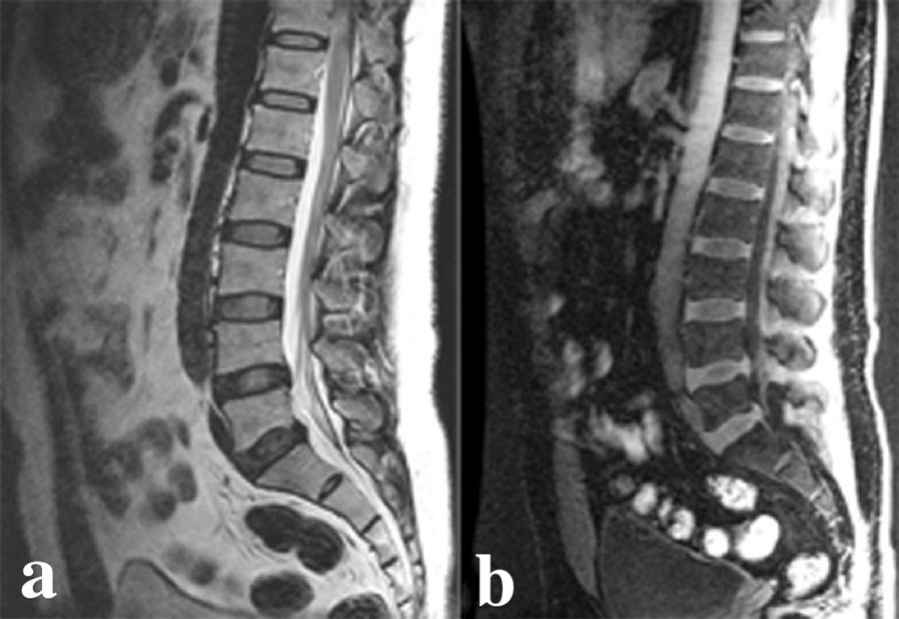
**a** Sagittal T2WI image; **b** sagittal 3D LAVA-Flex image. L1–2, L2–3, and L3–4 intervertebral discs (Pfirrmann grade II): the distinction of nucleus pulposus and annulus fibrosus was clear on T2WI and LAVA-Flex images. L4–5 (Pfirrmann grade IV) and L5–S1 intervertebral disc (Pfirrmann grade V): the distinction of nucleus pulposus and annulus fibrosus was unclear on T2WI image, and it was blurred or lost on 3D LAVA-Flex image

**Fig. 2:**
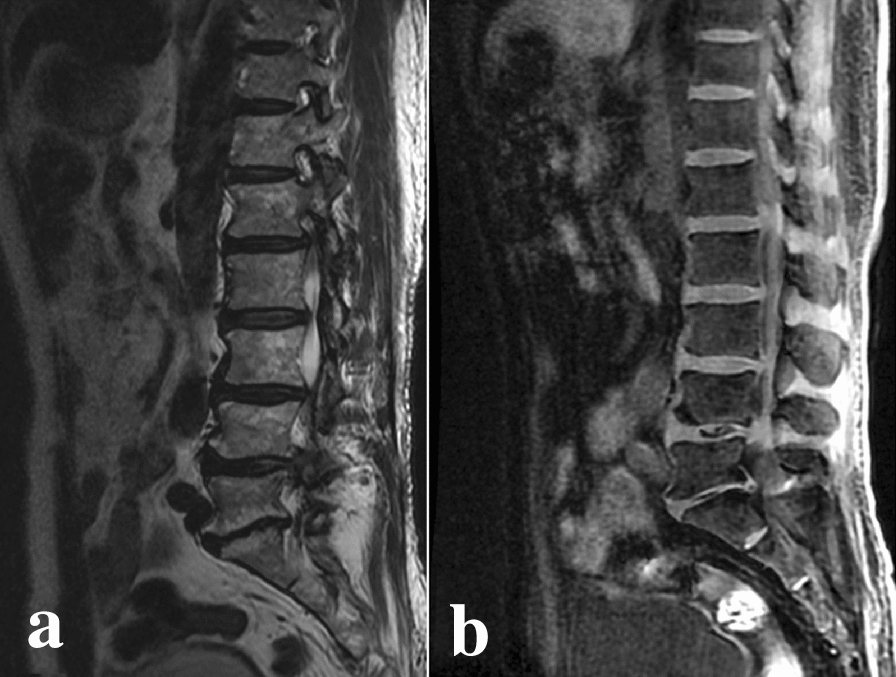
**a** Sagittal T2WI image; **b** sagittal 3D LAVA-Flex image. L4–5 and L5–S1 intervertebral space stenosis (Pfirrmann grade V): hypointense signal of the intervertebral disc appeared on T2WI image and on 3D LAVA-Flex image, linear hypointense signal shadow was present and the distinction of nucleus pulposus and annulus fibrosus was lost

(1) According to Pfirrmann classification, the distinction of nucleus pulposus and annulus fibrosus of grade I–II intervertebral disc was clear on T2WI images and the distinction was lost on T2WI images of grade III–IV intervertebral disc. On 3D LAVA-Flex images, the distinction of nucleus pulposus and annulus fibrosus was divided into two kinds: clear and lost distinction (Fig. 1). The presence of linear hypointense signal of nucleus pulposus on 3D LAVA-Flex images was evaluated (Fig. 2). (2) On 3D LAVA-Flex images, intervertebral disc space was divided into two kinds: normal and collapsed. (3) Lumbar intervertebral disc degeneration was divided into two grades according to the distinction of nucleus pulposus and annulus fibrosus, presence of linear hypointense signal of annulus fibrosus, and intervertebral disc space: I, clear distinction of nucleus pulposus and annulus fibrosus, absence of linear hypointense signal of annulus fibrosus, and narrowed or normal intervertebral disc space; II, unclear distinction of nucleus pulposus and annulus fibrosus, presence of linear hypointense signal of annulus fibrosus, narrowed or normal intervertebral disc space. (4) Virtual endoscopy for evaluation of intervertebral disc prolapse: 3D LAVA-Flex images of intervertebral disc were processed by virtual endoscopy on GE AW4.4 workstation (GE Medical Systems) and the anatomic relationship between degenerated intervertebral disc and intervertebral foramen.

### Image postprocessing

Raw data were transferred to AW4.4 workstation (GE Medical Systems) for comparison of identical positions between T2WI and 3D LAVA-Flex images to observe degenerated disc and intervertebral foramen.

### Statistical analysis

General descriptive statistics were expressed as mean ± SD. Chi-square test was used to examine differences of qualitative data between T2WI and 3D LAVA-Flex images. A level of *P* < 0.05 was considered statistically significant.

## Results

### 3D LAVA-Flex image features of normal intervertebral discs

**Fig. 3:**
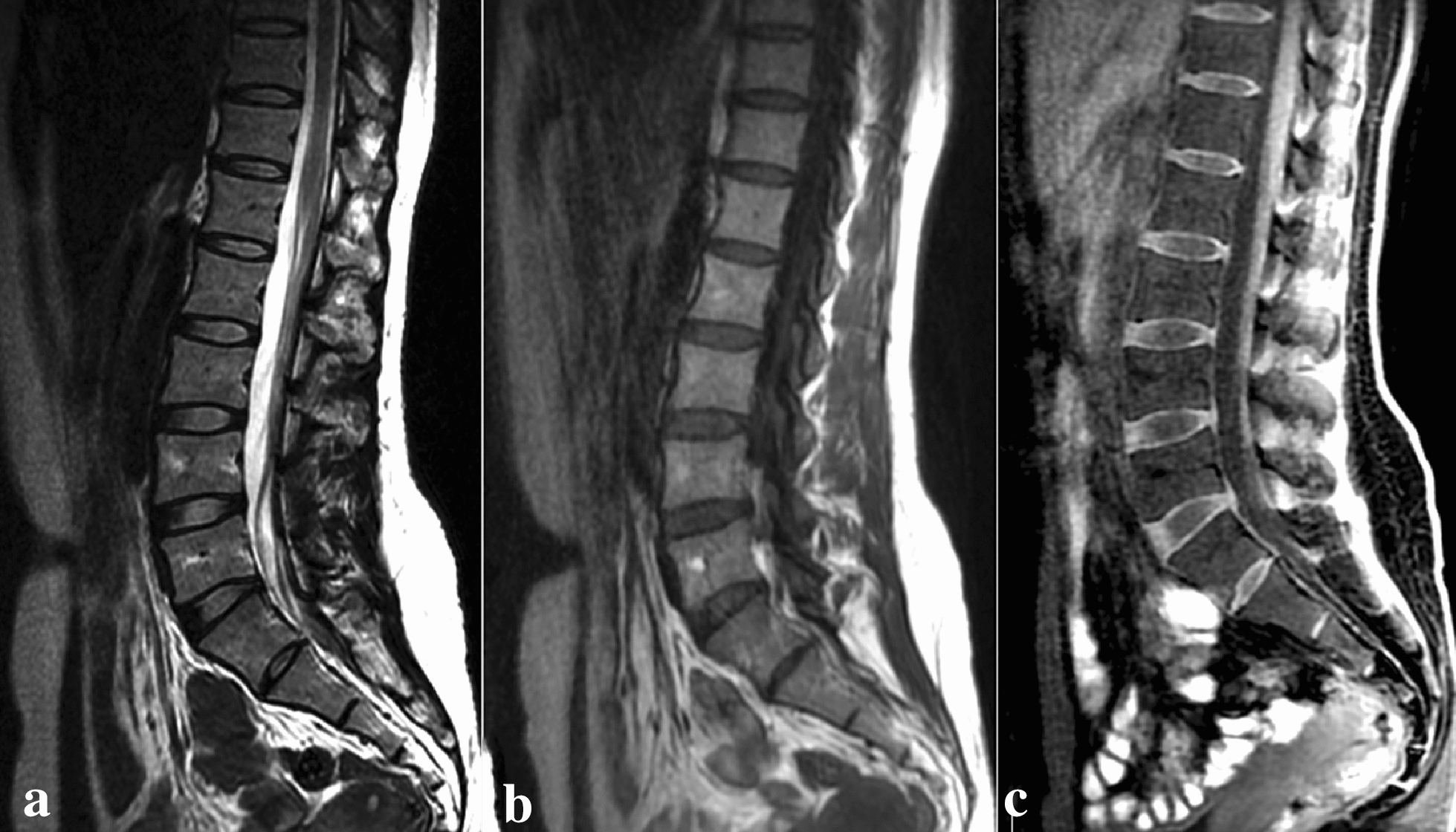
Normal intervertebral disc. Sagittal T2WI (**a**), T1WI (**b**) and 3D LAVA-Flex images (**c**)

L1–2 and L5–S1 segments of each patient were observed. Among 225 intervertebral discs (45 patients × 5), 133 were normal on 3D LAVA-Flex images, with the features of homogeneous intermediate–intense signal of the nucleus pulposus, complete ring-shaped hyperintense signal of annulus fibrosus and clear distinction of nucleus pulposus and annulus fibrosus (Fig. 3).

### Comparison of degenerated intervertebral discs on T2WI and 3D LAVA-Flex images

On 3D LAVA-Flex images, the distinction of nucleus pulposus and annulus fibrosus was clear in 133 intervertebral discs and it was unclear in 92 intervertebral discs. On T2WI images, the distinction of nucleus pulposus and annulus fibrosus was clear in 115 intervertebral discs and it was unclear in 110 intervertebral discs. Chi-square test showed that there was no significant difference in number of degenerated intervertebral disc between T2WI and 3D LAVA-Flex images (χ^2^ = 2.910, *P* = 0.088) (Table 1). On 3D LAVA-Flex images, linear hypointense signal of annulus fibrosus was present in 4 out of 134 intervertebral discs with normal disc space (4/134, 2.98%) and in 65 out of 91 intervertebral discs with collapsed disc space (65/91, 71.43%). Chi-square test showed that there was significant difference between normal disc space and collapsed disc space (*P* = 0.000) (Table 2). According to 3D LAVA-Flex classification, 116 intervertebral discs were classified as grade I and 109 as grade II. According to T2WI-based Pfirrmann classification, 119 were classified as grade I–II and 106 as grade III–V. Kappa value for each classification was 0.902 (*P* = 0.000) and therefore the outcomes of 3D LAVA-Flex classification and T2WI-based Pfirrmann classification were consistent (Table 3).

**Table 1:** Comparison of distinction of nucleus pulposus and annulus fibrosus between 3D LAVA-Flex and T2WI images

T2WI	3D LAVA-Flex	
	Clear distinction	Unclear distinction
Clear distinction (I–II)	108	7
Unclear distinction (III–V)	25	85

χ^2^ = 2.910, *P* = 0.088

**Table 2:** Correlation of linear hypointense signal on 3D LAVA-Flex image with intervertebral disc necrosis

	Linear hypointense signal	Homogenous signal
Normal intervertebral disc space I–II	4	130
Collapsed intervertebral disc space III–V	65	26

Kappa = 0.902, *P* = 0.000

**Table 3:** A comparison of 3D LAVA-Flex classification with T2WI Pfirrmann grade

Pfirrmann grade	3D LAVA-Flex classification	
	I	II
I–II	112	7
III–V	4	102

Kappa = 0.902, *P* = 0.000

### Virtual endoscopy

**Fig. 4:**
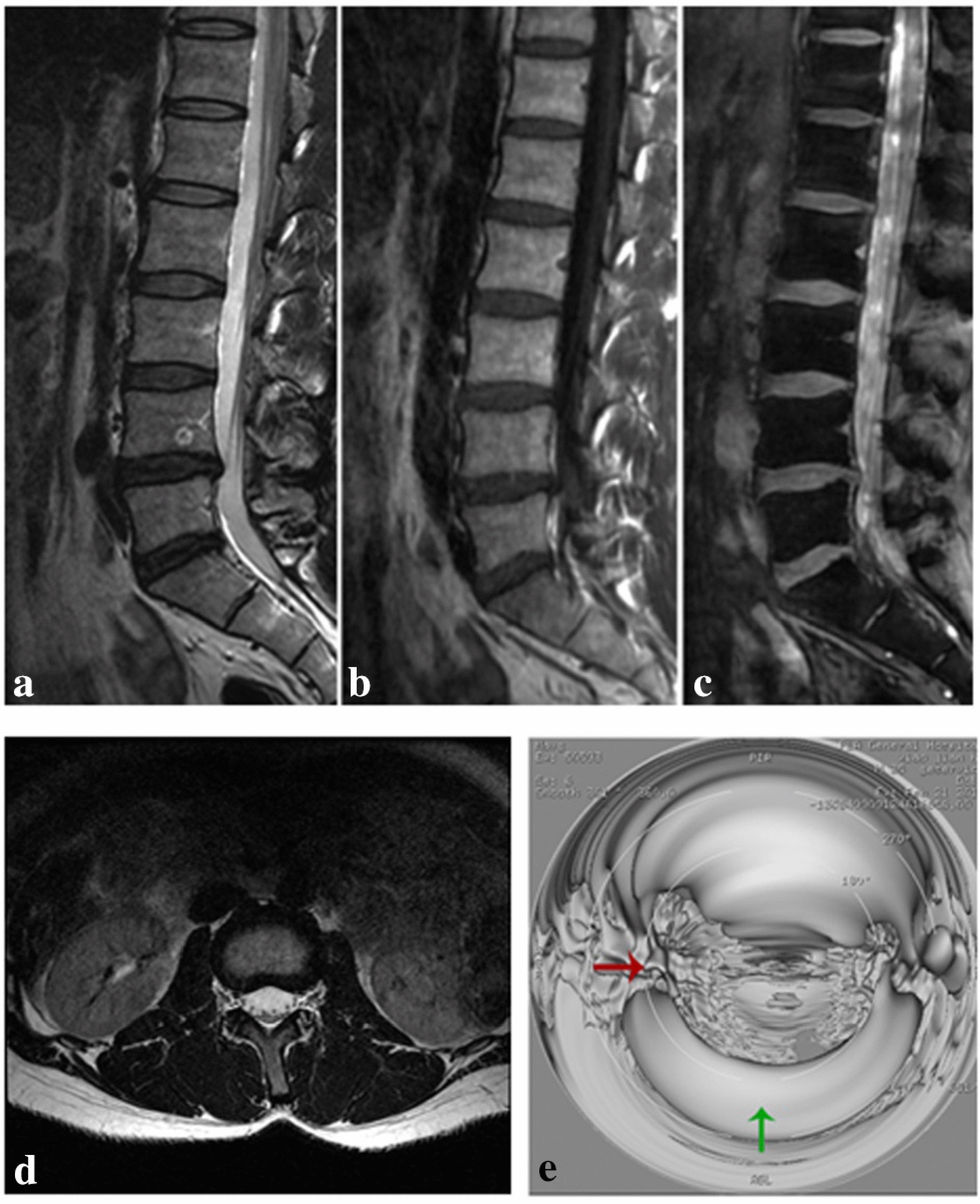
Images of a 40-year-old male who had suffered from low back pain for 2 years. Sagittal T2WI (**a**), T1WI (**b**), 3D LAVA-Flex (**c**), and horizontal T2WI (normal) images of L3–4 segments (**d**). Virtual endoscopy (**e**) finding of normal L3-4 segments. In **e**, red arrow indicates right-sided intervertebral foramen, and green arrow normal intervertebral disc

**Fig. 5:**
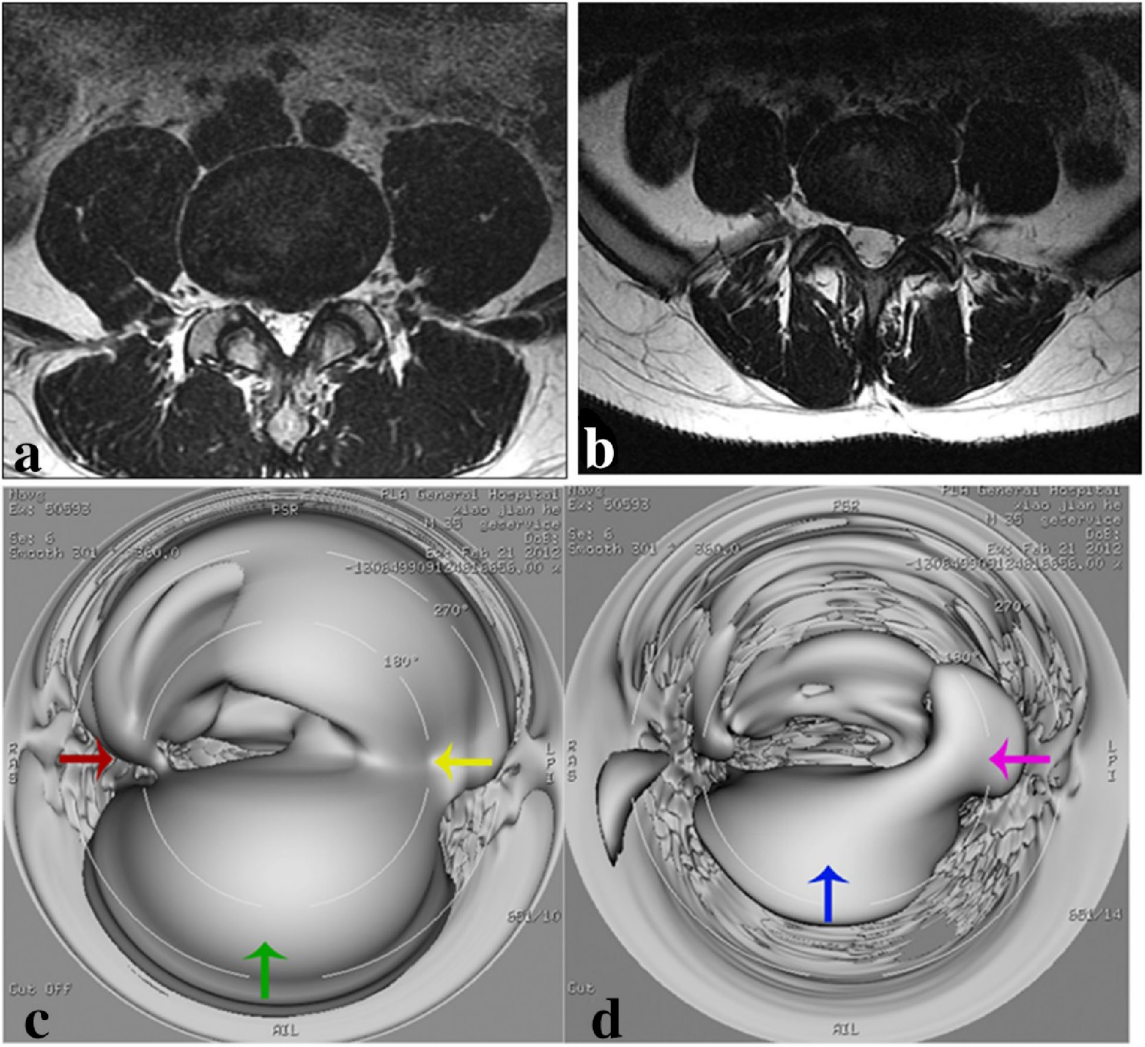
Virtual endoscopy images of L4–5 and L5–S1 segment intervertebral disc prolapse in a 40-year-old male who had suffered from low back pain for 2 years. L4–5 segment intervertebral disc (**a**, **c**). Red arrow indicates right-sided intervertebral foramen, green arrow globular protruded intervertebral disc and yellow arrow compressed left-sided intervertebral foramen. L5–S1 segment intervertebral disc (**b**, **d**). Blue arrow indicates protruded intervertebral disc and pink arrow compressed left-sided intervertebral foramen

3D LAVA-Flex image-based virtual endoscopy was performed in 45 patients and it well displayed the anatomic relationship between degenerated intervertebral disc and intervertebral foramen (Figs. 4, 5).

## Discussion

Degeneration and prolapse of lumbar intervertebral disc are common causes of low back and leg pains which often need surgical treatment [10]. The intervertebral disc is composed of nucleus pulposus, annulus fibrosus and cartilage endplate. Excessive loading of intervertebral disc caused by genetic, biomechanical, biochemical, cytochemical and psychological factors will first cause damage to the water-containing jelly-like nucleus pulposus, leading to decreased activity of cells in the nucleus pulposus, reduced generation of the proteoglycan that maintains tissue water content, decreased size of proteoglycan molecule, and reduced water content in the nucleus pulposus [2].Traumatic intervertebral disc lesions can result in rapid biochemical changes in the nucleus pulposus [3]. Therefore, dehydration of the nucleus pulposus occurs in a short period after trauma. Degeneration of the intervertebral disc indicates the specific or malignant changes in intervertebral disc component, structure and function. Different from degeneration, aging is only a risk factor for intervertebral disc prolapse, and it possibly leads to biochemical changes of nucleus pulposus and therefore it is not an absolute cause of low back pain. Various factors including intervertebral disc prolapse and degeneration (annulus fibrosus, cartilage endplate, ligament and vertebral joints) can cause intervertebral foramina narrowing and nerve root compression, which correspond to some symptoms of low back and legs [11].

Diagnosis of low back and leg pain and selection of proper treatment methods depend to a large extent on imaging examination. X-ray and CT are conventional imaging methods and they are inferior to MRI with considerations of tissue resolution and safety, and in particularly use to determine the causes of spinal cord or nerve root compression [12]. With development of computer technique and imaging methods, some novel techniques have been developed. LAVA-Flex is 3D spoiled gradient echo pulse sequence that is recently equipped with water–fat separation technique, which betters tissue suppression. This technique has been used for screening abdominal and pelvic cavity lesions because it can help observe the anatomic structure of lesioned and normal tissue from different views and acquire stable water-only and fat-only images in one single acquisition in a short time period [6, 7].

Conventional T2WI is a classical lesion-visualizing sequence. It can help visualize the increased hydrogen protons in some disorders, such as inflammation and tumor. Under the condition of dehydration caused by intervertebral disc degeneration, the nucleus pulposus and annulus fibrosus cannot be further distinguished by T2WI images because the number of hydrogen protons is decreased at this time. 3D LAVA-Flex, as T1WI for structure observation, can clearly display annulus fibrosus and the greatly dehydrated nucleus pulposus. Virtual endoscopy is an advanced 3D imaging technique that can objectively reflect intervertebral foramen stenosis caused by intervertebral disc prolapse, which is a technique breakthrough of MRI post-processing and is a beneficial supplement of conventional MRI technique.

Pfirrmann grading has been widely accepted to grade intervertebral disc lesions [13]. It can help observe the distinction of nucleus pulposus and annulus fibrosus on sagittal T2WI image. It consists of 5 grades: grades I–II, clear distinction of nucleus pulposus and annulus fibrosus; grades III–IV, lost distinction of nucleus pulposus and annulus fibrosus. According to 3D LAVA-Flex images, the distinction of nucleus pulposus and annulus fibrosus can be assigned to two types: clear and lost. 3D LAVA-Flex can well visualize the distinction of nucleus pulposus and annulus fibrosus and help evaluate linear hypointense signal of nucleus pulposus. Our results showed that 3D LAVA-Flex is consistent with conventional T2WI, provides a novel method of evaluating intervertebral disc lesions and shows great potential in the study of intervertebral disc abnormalities.

3D LAVA-Flex images show hypointense signal of the intervertebral disc, which occurs possibly because of nucleus pulposus fibrosis, annulus fibrosus fragmentation and calcification. But the precise pathophysiological mechanism needs further investigation. Limitations of this study include lack of pathological control, slightly small sample size, and lack of postoperative follow up.

## Conclusions

3D LAVA-Flex can be used to effectively evaluate the distinction of nucleus pulposus and annulus fibrosus of degenerated intervertebral disc and visualize linear hypointense signal of the degenerated intervertebral disc. 3D LAVA-Flex can help to simply and accurately grade the intervertebral disc lesions. Virtual endoscopy can be used to evaluate the anatomic relationship between degenerated intervertebral disc and intervertebral foramen.

## Data Availability

The datasets collected and analyzed during the current study are available from the corresponding author upon reasonable request.
